# Pushing the Limit for Marginal Grafts in the Era of Machine Perfusion: A Tale of HOPE From a Leading Italian Institution

**DOI:** 10.1111/aor.70137

**Published:** 2026-04-28

**Authors:** Gerti Dajti, Giuliana Germinario, Enrico Prosperi, Guido Fallani, Chiara Bonatti, Edoardo Prosperi, Antonio Siniscalchi, Maria Cristina Morelli, Antonietta D’ Errico, Matteo Serenari, Massimo Del Gaudio, Chiara Zanfi, Federica Odaldi, Lorenzo Maroni, Andrea Laurenzi, Matteo Cescon, Matteo Ravaioli

**Affiliations:** ^1^ Department of Medical and Surgical Sciences University of Bologna Bologna Italy; ^2^ IRCCS Azienda Ospedaliero‐Universitaria di Bologna Bologna Italy

## Abstract

**Background:**

The advent of machine perfusion (MP) has significantly improved post‐liver transplantation (post‐LT) outcomes, potentially enabling the use of increasingly marginal grafts and expanding the organ donor pool.

**Methods:**

We present a retrospective cohort study of consecutive adult patients who underwent LT between 2018 and 2023 at a leading Italian institution. The objective was to evaluate outcomes following the use of hypothermic oxygenated perfusion (HOPE) in high‐risk grafts.

**Results:**

A total of 507 patients were included in the final analysis, of whom 420 (83%) received extended criteria donor (ECD) grafts. Among ECD grafts, 62 (15%) were from donation after circulatory death (DCD) donors, and 64 (15%) were previously discarded by other centers. HOPE was applied in 248 (49%) cases. Recipients in the HOPE group experienced significantly lower rates of early allograft dysfunction (EAD) (20% vs. 32%, *p* = 0.007), primary nonfunction (2% vs. 7%, *p* = 0.017), and severe postoperative complications (Clavien–Dindo grade ≥ 3b) (19% vs. 28%, *p* = 0.026). Notably, marginal grafts treated with HOPE achieved survival outcomes comparable to those of standard risk.

**Conclusions:**

HOPE is associated with improved outcomes in LT using ECD grafts and can enable the safe use of higher‐risk organs with acceptable results when performed in experienced centers.

## Graphical abstract

HOPE has improved outcomes in ECD liver transplantation and allowed the safe utilization of increasingly marginal grafts with favorable results.

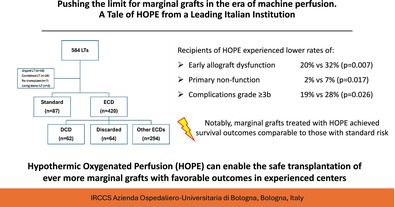

**Abbreviations**
BMI: body mass index
CIT: cold ischemia time
DCD: donor from circulatory death
DRI: donor risk index
EAD: early allograft dysfunction
ECD: extended criteria donors
HOPE: hypothermic oxygenated perfusion
IRI: ischemic reperfusion injury
LT: liver transplantation
MELD: model for end‐stage liver disease
MP: machine perfusion
POD: postoperative day
SCS: static cold storage
WIT: warm ischemia time

## 1 Introduction

The increasing demand for liver transplantation (LT) has led to the broader use of marginal grafts which, however, are associated with a higher risk of graft dysfunction and poorer outcomes. Optimal management at all stages of the LT process is essential to mitigate graft injury and improve post‐transplant outcomes.

In recent years, the advent of machine perfusion (MP) technologies has been a major innovation in the field, particularly for extended criteria donors (ECD). By attenuating ischemic‐reperfusion injury (IRI) [1], MP has been shown to reduce rates of early allograft dysfunction (EAD), postoperative complications—including nonanastomotic biliary strictures—and graft loss [2, 3, 4]. Furthermore, MP enables viability assessment of grafts that would otherwise be discarded, thereby potentially expanding the transplantable organs pool [5].

The Italian transplant landscape is characterized by a relatively limited donor supply and prolonged waiting list times, despite a recent improvement in donation trends. The use of marginal grafts, including DCDs, has therefore become a cornerstone of routine clinical practice. The 20 min no‐touch period, mandate by Italian law, adds another layer of complexity and risk for DCDs donors. In this study, we present our center's experience with HOPE and its impact on outcomes in high‐risk liver grafts.

## 2 Materials and Methods

We conducted a retrospective cohort study of consecutive adult patients undergoing LT at our Institution between January 1, 2018 and December 31, 2023. Exclusion criteria included (i) urgent LTs, (ii) retransplantations, (iii) combined transplantations and (iv) living donor LTs. The institutional Ethics Committee waived approval due to the retrospective nature of the study. The study was conducted in accordance with STROBE guidelines [6].

ECD donors were defined based on the following criteria: (a) hemodynamic instability; (b) donor age > 65 years; (c) body mass index (BMI) > 30 kg/m^2^; (d) serum bilirubin > 3 mg/dL; (e) aspartate or alanine aminotransferase (AST or ALT) > 3 times the upper reference threshold; (f) serum sodium > 165 mmol/L; (g) intensive care unit stay > 7 days; (h) macro steatosis > 40%; or (i) cold ischemia time (CIT) > 12 h. Discarded grafts were defined as those accepted and transplanted at our Institution after initially being offered and declined by all other centers within the national allocation system. Donor risk index (DRI) was calculated as proposed by *Feng et al.* [7].

In Italy, normothermic regional perfusion (NRP) and HOPE are mandated for all donation after circulatory death (DCD). In other cases, HOPE was used within clinical trials or based on surgeon's discretion.

HOPE was performed using the Vitasmart device (Bridge to Life, USA) in blood‐free preservation technique. Briefly, graft perfusion began in the operating room during back‐table preparation, including an initial flushing phase (30 mL/min) to remove waste products and residual microthrombi, followed by continuous perfusion until organ implantation. Recipient warm ischemia time (rWIT) was defined as the time interval from graft removal from cold preservation to portal reperfusion.

Postoperative complications were assessed at 6 months after LT. EAD was defined as the presence of one of the following criteria (i) serum bilirubin > 10 mg/dL on postoperative day (POD) 7; (ii) international normalized ratio (INR) > 1.6 on POD7; or (iii) AST/ALT > 2000 U/L within the first seven postoperative days [8]. Complications were described according to the Clavien–Dindo classification and the Comprehensive Complication Index [9].

Statistical analysis was performed using the STATA software version 18.0 (TX, USA). Parametric and nonparametric tests were used as appropriate to compare groups. Continuous variables are reported as median values and range. Kaplan–Meier curves were employed for survival analysis. Multivariate models were built in a backward fashion including variables with a *p*‐value of at most 0.100 in the univariate analysis. The threshold for statistical significance was set at *p* < 0.05.

## 3 Results

**FIGURE 1:**
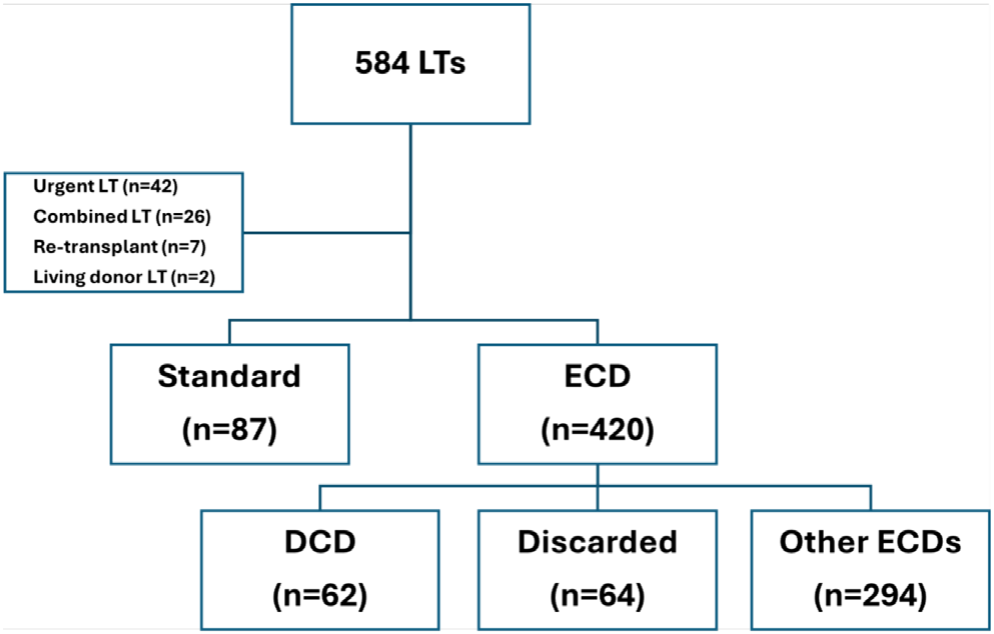
Study design and cohort selection flowchart. [Color figure can be viewed at wileyonlinelibrary.com]

A total of 507 patients undergoing LT during the study period were included in the final cohort (Figure 1), of which 420 (83%) received grafts from ECD donors. Baseline donor characteristics and postoperative outcomes by donor type (standard criteria vs. ECD) are summarized in Table 1. ECD recipients had lower MELD at transplantation and were more likely to receive HOPE‐treated grafts. There were no significant differences in postoperative complication rates and graft survival between the two groups.

**TABLE 1:** Baseline characteristics and outcomes.

	All patients (*n* = 507)	Standard (*n* = 87)	ECD (*n* = 420)	*p*
**Recipient**				
Age (years)	59 (21–75)	59 (21–74)	59 (22–75)	0.8
Male gender	345 (68%)	61 (70%)	284 (68%)	0.7
BMI (kg/m^2^)	25.0 (13.0–42.0)	25.0 (17.0–39.0)	25.0 (13.0–42.0)	0.2
MELD at transplant	14 (6–34)	16 (6–34)	14 (6–34)	0.054
Portal thrombosis	98 (19%)	19 (22%)	79 (19%)	0.5
HCC	228 (44%)	33 (38%)	195 (46%)	0.1
**Donor**				
Age (years)	67 (7–94)	52 (12–64)	71 (7–94)	0.000
Male gender	277 (55%)	44 (51%)	233 (55%)	0.4
BMI (kg/m^2^)	25.5 (15.8–50.8)	24.7 (17.5–29.8)	25.8 (15.8–50.8)	0.001
Macrosteatosis (%) (*n* = 379)	2 (0–40)	0 (0–20)	2 (0–40)	0.002
Donor Risk Index	1.766 (0.888–2.992)	1.423 (0.906–2.182)	1.836 (0.888–2.992)	0.000
Cold Ischemia time (min)	390 (220–1090)	395 (270–640)	390 (220–1090)	0.5
**Transplantation and outcomes**				
Operation time (min)	480 (290–1155)	495 (315–780)	480 (290–1155)	0.3
HOPE	248 (49%)	16 (18%)	232 (55%)	0.000
Reperfusion syndrome (*n* = 419)	185 (44%)	26 (38%)	159 (45%)	0.3
EAD	127 (25%)	22 (25%)	105 (25%)	1.0
PGNF	19 (4%)	1 (1%)	18 (4%)	0.2
Complications				
Infective (*n* = 499)	192 (38%)	29 (34%)	163 (39%)	0.4
Pulmonary (*n* = 419)	44 (11%)	13 (19%)	31 (9%)	0.1
CVVH (*n* = 499)	53 (11%)	10 (12%)	43 (10%)	0.7
Ascites (*n* = 496)	153 (31%)	22 (26%)	131 (32%)	0.3
Biliary (*n* = 496)	44 (9%)	6 (7%)	38 (9%)	0.5
Vascular (*n* = 494)	36 (7%)	5 (6%)	31 (8%)	0.6
Cardiologic (*n* = 415)	43 (10%)	4 (6%)	39 (11%)	0.2
Reintervention (*n* = 494)	68 (14%)	14 (17%)	54 (13%)	0.4
Acute rejection (*n* = 499)	23 (5%)	3 (4%)	20 (5%)	0.6
CCI	29.9 (0.0–100.0)	29.6 (0.0–100.0)	29.6 (0.0–100.0)	0.8
Clavien–Dindo ≥ 3b	121 (24%)	24 (28%)	97 (23%)	0.4
Graft loss at 1 year	54 (11%)	6 (7%)	48 (11%)	0.2

Abbreviations: BMI, Body Mass Index; CCI,Comprehensive Complications Index; EAD, Early allograft dysfunction; ECD,Extended Criteria Donor; HCC, Hepatocellular carcinoma; HOPE, Hypothermic oxygenated perfusion; MELD, Model for End‐Stage Liver Disease; PGNF, Primary graft non function.

Among ECD donors, 62 grafts were from DCD donors, and 64 grafts had been previously discarded from other centers (Table 2). Primary reasons for discard included macrosteatosis > 40% (*n* = 7), advanced donor age > 85 years (*n* = 2), and elevated donor risk due to infective (*n* = 24), oncological (*n* = 10) or medical reasons (*n* = 8), vascular, mismatch and other causes (*n* = 13) [10]. No graft was discarded due to adverse finding during MP. DCD and discarded grafts were associated with lower MELD at transplantation and donor age. Recipients of discarded grafts had higher donor BMI, macrosteatosis at biopsy and longer ischemia time. Postoperative complications and graft survival were similar among the groups with two exceptions: EAD developed less frequently in the DCD group (13% vs. 27%, *p* = 0.017), whereas hepatic artery thrombosis was more common among DCD and discarded ECD grafts (vs. others 3% vs. 0%, *p* = 0.014).

**TABLE 2:** Patients' features and outcomes by type of graft for ECD donors.

	Discarded (*n* = 64)	DCD (*n* = 62)	Other ECD (*n* = 294)	*p*
**Recipient**				
Age (years)	59 (41–73)	59 (38–73)	59 (21–75)	0.7
Male gender	41 (64%)	43 (69%)	200 (68%)	0.8
BMI (kg/m^2^)	26.0 (19.5–40.1)	25.0 (18.4–42)	25.0 (13.0–40.4)	0.3
MELD at transplant	14 (6–29)	12 (6–31)	15 (6–34)	0.023
Portal thrombosis	9 (14%)	10 (16%)	60 (20%)	0.4
HCC	32 (50%)	30 (48%)	133 (45%)	0.7
**Donor**				
Age (years)	63 (7–94)	69 (25–86)	73 (12–89)	0.000
Male gender	38 (59%)	36 (58%)	159 (54%)	0.7
BMI (kg/m^2^)	27.8 (15.8–45.0)	25.8 (18.6–49.0)	25.3 (17.2–50.8)	0.014
Macrosteatosis (%)	6.5 (0–40)	0 (0–30)	2 (0–40)	0.006
Donor risk index	1.650 (0.936–2.276)	2.624 (1.611–2.992)	1.786 (0.888–2.119)	0.000
Cold ischemia time (min)	458 (325–1070)	360 (220–600)	390 (240–1090)	0.000
**Transplantation and outcomes**				
Operation time (min)	450 (290–600)	445 (335–895)	485 (300–1155)	0.006
HOPE	39 (61%)	62 (100%)	131 (45%)	0.000
Reperfusion syndrome	23 (44%)	19 (40%)	117 (46%)	0.8
EAD	15 (23%)	8 (13%)	82 (28%)	0.044
PGNF	4 (6%)	2 (3%)	12 (4%)	0.7
Complications				
Infective	28 (45%)	23 (37%)	112 (39%)	0.6
Pulmonary	5 (10%)	4 (9%)	22 (9%)	0.9
CVVH	6 (10%)	7 (11%)	30 (10%)	0.9
Ascites	16 (26%)	18 (29%)	97 (34%)	0.4
Biliary	5 (8%)	6 (10%)	27 (9%)	0.9
Vascular	3 (5%)	5 (8%)	23 (8%)	0.7
Cardiologic	4 (8%)	3 (6%)	32 (13%)	0.3
Reintervention	10 (16%)	6 (10%)	38 (13%)	0.6
Acute rejection	0 (0%)	2 (3%)	18 (6%)	0.096
CCI	35.0 (0.0–100)	29.6 (0.0–100)	29.6 (0.0–100)	0.2
Clavien–Dindo ≥ 3b	18 (18%)	14 (23%)	65 (22%)	0.6
Graft loss at 1 year	9 (14%)	9 (15%)	30 (10%)	0.5

Abbreviations: BMI, Body Mass Index; CCI, Comprehensive Complications Index; DCD, Donor from cardiac death; EAD, Early allograft dysfunction; ECD, Extended Criteria Donor; HCC, Hepatocellular carcinoma; HOPE, Hypothermic oxygenated perfusion; MELD, Model for End‐Stage Liver Disease; PGNF, Primary graft non function.

**FIGURE 2:**
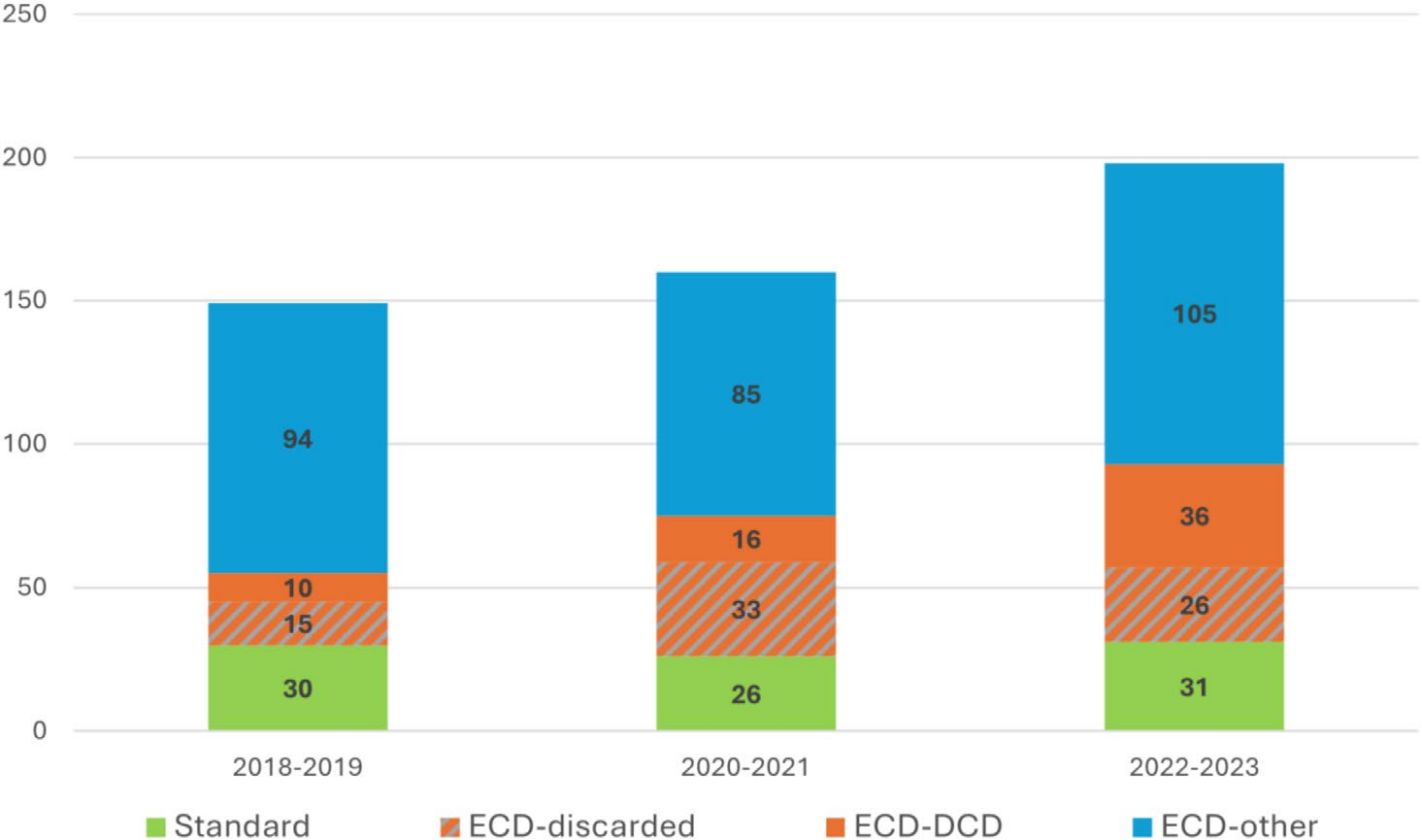
Type of donor distribution divided by time period. [Color figure can be viewed at wileyonlinelibrary.com]

**FIGURE 3:**
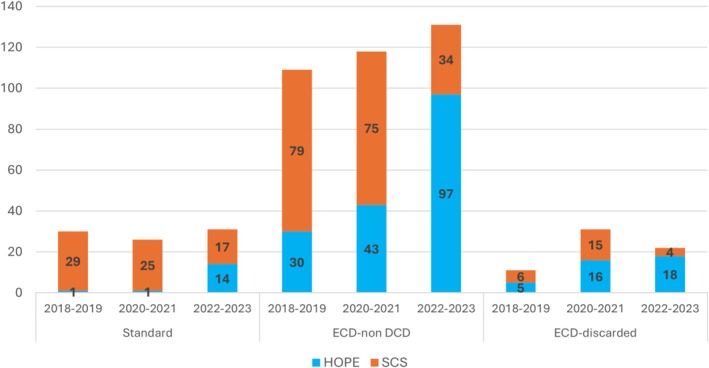
HOPE use divided by time period and time. [Color figure can be viewed at wileyonlinelibrary.com]

The distribution of utilized grafts by donor type changed over time with a stable increasing trend for ECD donors, including DCD and discarded grafts. (Figure 2) Similarly, HOPE was applied significantly more with time (*r* ^*2*^ 0.173, *p* < 0.001), particularly among ECD donors (Figure 3). Overall, 248 (49%) patients received grafts treated with HOPE before LT, with a median perfusion time of 135 min. Baseline features and outcomes divided by the preservation technique in ECD grafts are detailed in Table 3. As expected, HOPE‐treated grafts had significantly higher DRI but were associated with lower rates of EAD (20% vs. 32%, *p* = 0.007), primary graft nonfunction (2% vs. 7%, *p* = 0.017), and postoperative complications grade 3b or higher according to Clavien–Dindo (19% vs. 28%, *p* = 0.026). Additionally, logistic regression analysis was performed to identify risk factors for EAD in ECD donors (Table S1). At multivariate, HOPE and CIT were the only independent factors for EAD development (OR 0.504, 95% CI 0.319–0.795 and OR 1.004, 95% CI 1.002–1.006, respectively).

**TABLE 3:** Patients' features and outcomes (SCS vs. HOPE) for ECD donors.

	SCS (*n* = 188)	HOPE (*n* = 232)	*p*
**Recipient**			
Age (years)	58 (22–73)	59 (21–75)	
Male gender	123 (65%)	161 (70%)	0.4
BMI (kg/m^2^)	25.0 (13.0–38.0)	25.7 (17.0–42.0)	0.5
MELD at transplant	15 (6–34)	13 (6–34)	0.2
Portal thrombosis	31 (16%)	48 (21%)	0.3
HCC	95 (51%)	100 (43%)	0.1
**Donor**			
Age (years)	69 (18–89)	72 (7–94)	0.2
Male gender	104 (55%)	129 (56%)	0.9
BMI (kg/m^2^)	26.0 (17.2–46.9)	25.5 (15.8–50.8)	0.9
Macrosteatosis (%) (*n* = 335)	2 (0–40)	3 (0–40)	0.9
Donor risk index	1.766 (0.936–2.119)	1.908 (0.888–2.992)	0.000
Cold Ischemia time (min)	400 (240–720)	380 (220–1090)	0.2
**Transplantation and outcomes**			
Operation time (min)	480 (290–1155)	480 (295–895)	0.2
Reperfusion syndrome (*n* = 352)	88 (48%)	71 (43%)	0.3
EAD	59 (32%)	49 (20%)	0.007
PGNF	13 (7%)	5 (2%)	0.017
Complications			
Infective (*n* = 414)	74 (40%)	89 (39%)	0.8
Pulmonary (*n* = 352)	18 (10%)	13 (8%)	0.5
CVVH (*n* = 414)	22 (12%)	21 (9%)	0.3
Ascites (*n* = 412)	60 (33%)	71 (31%)	0.7
Biliary (*n* = 411)	21 (12%)	17 (7%)	0.2
Vascular (*n* = 409)	16 (9%)	15 (7%)	0.4
Cardiologic (*n* = 349)	26 (14%)	13 (8%)	0.1
Reintervention (*n* = 411)	26 (14%)	28 (12%)	0.6
Acute rejection (*n* = 414)	12 (6%)	8 (3%)	0.2
CCI	30.8 (0.0–100.0)	29.6 (0.0–100.0)	0.1
Clavien–Dindo ≥ 3b	53 (28%)	44 (19%)	0.026
Graft loss at 1 year	22 (12%)	26 (11%)	0.9
DCD	—	9 (15%)	—
Discarded	4 (16%)	5 (13%)	0.7
Other	18 (11%)	12 (9%)	0.6

Abbreviations: BMI, Body Mass Index; CCI, Comprehensive Complications Index; DCD, Donor from cardiac death; EAD, Early allograft dysfunction; ECD, Extended Criteria Donor; HCC, Hepatocellular carcinoma; HOPE, Hypothermic oxygenated perfusion; MELD, Model for End‐Stage Liver Disease; PGNF, Primary graft non function; SCS, Static cold storage.

**FIGURE 4:**
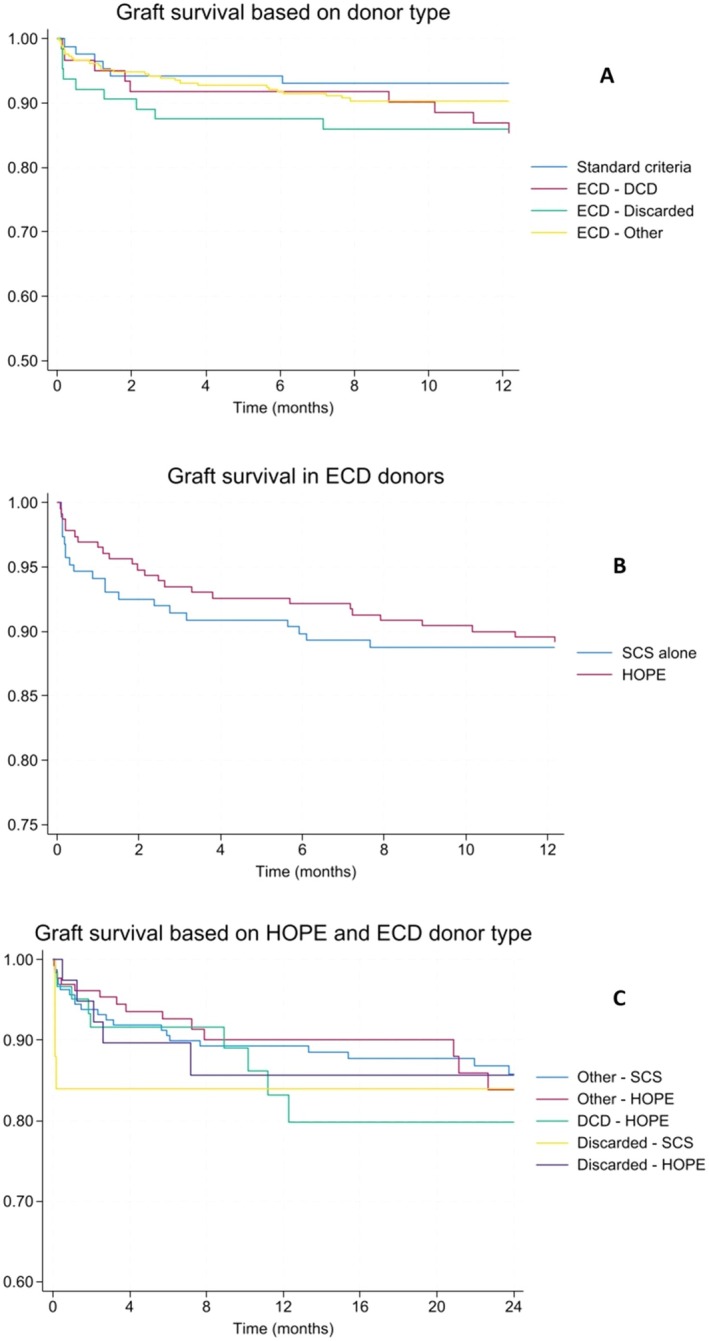
Kaplan–Meier curves divided by type of donor (A), HOPE in ECD donors (B), HOPE and donor type in ECD (C). [Color figure can be viewed at wileyonlinelibrary.com]

With a median follow‐up of 1.4 years, graft survival at 12 months was comparable across donor groups: standard criteria (93%), DCD (85%), discarded (86%) and other ECD (90%) (*p* = 0.4). Subgroup survival by donor and preservation method also showed no significant differences (Table S2; Figure 4).

## 4 Discussion

Organ shortage remains a challenge for Transplant Centers worldwide despite increasing donation and transplantation rates. Marginal grafts, including DCDs, are often used to address the discrepancy, as confirmed by US and European data [11, 12]. Italy represents a clear example, as historically, ECDs represent the majority of donors due to limited organ availability and long waiting lists. However, recent official data confirm a steady rise in donations, including DCDs, while maintaining a relatively low discard rate (11% and 4% of all offered and procured organs, respectively) [13].

Our findings demonstrate how the adoption of MP, particularly HOPE, combined with optimized surgical protocols, has enabled successful use of increasingly marginal grafts. In spite of their inherent risks, ECDs can yield excellent outcomes when carefully selected and managed.

Although outcomes with marginal grafts have improved, they are still preferentially assigned to recipients with well compensated liver disease. Our center adopts the same policy as shown by the lower MELD in the DCD and discarded groups. The lack of clear and objective guidelines makes the experience of the transplantation team crucial at this phase.

IRI leads to worse EAD and graft survival rates and is strictly related to warm and CITs [14]. Warm ischemia occurs during both procurement (donor WIT [dWIT]) and organ implantation (recipient WIT [rWIT]). Donor WIT is strongly associated with graft injury for DCD donors and post‐LT outcome. Despite different criteria being adopted among countries to either determine dWIT or viability for LT [15], it is generally accepted that dWIT longer than 30 min is associated with significantly worse outcomes. The mandatory 20‐min no‐touch period for DCD donors in Italy results in increased dWIT and, consequently, a higher risk to the graft. However, careful multidisciplinary management during procurement, together with team experience, is essential to limit dWIT and keep it within acceptable viability thresholds. Our results, consistent with other Italian experiences, demonstrate that these measures—along with the well‐established role of MP—can lead to satisfactory LT outcomes despite the higher dWIT compared with other countries.

Recipient WIT coincides with the implantation time. Proven and standardized surgical techniques have limited the variability of WIT; however, extensive rWIT has been associated with higher graft injury and dysfunction [16, 17].

CIT has a long‐established association with IRI, graft injury, and postoperative complications. Our results confirmed CIT as an independent risk factor for EAD in ECD grafts. Although surgery time can be influenced by multiple factors, it was shorter in the discarded group, highlighting the priority of keeping WIT and CIT to a minimum in order to contain IRI and the risk of post‐LT complications.

However, the most relevant novelty affecting LT in recent years is the introduction of MP. MP can reduce graft injury and prevent post‐LT complications, which has allowed to further push the boundaries for viability especially for high‐risk grafts. The three main techniques to date are NRP, hypothermic oxygenated perfusion (HOPE), and normothermic machine perfusion NMP [15]. Abdominal or thoraco‐abdominal NRP is an in situ perfusion technique based on extracorporeal membrane oxygenation (ECMO) technology. NRP's primary impact is observed for DCD donors due to its ability to limit graft damage associated with functional dWIT and reduce post‐LT complications [18, 19]. Due to the higher no‐touch period (20 min) defined by Italian law, NRP is mandatory and was applied to all DCD grafts in our cohort.

NMP and HOPE represent the two main ex‐situ perfusion techniques. Well‐supported evidence based on randomized controlled trials (RCT) has confirmed their ability to reduce rates of EAD, biliary complications, including nonanastomotic strictures, and to improve graft survival in ECD grafts [2, 3, 4, 20, 21, 22, 23, 24]. In our Center, HOPE is performed routinely for DCD grafts and majority of other ECD donors. HOPE has been increasingly implemented, particularly following favorable RCT results published by the Authors in 2022 [4]. Such trend was observed among all different types of grafts, including standard criteria grafts. In fact, 85% of all transplanted grafts in the last year of the study were perfused with HOPE before LT (96% if considering only ECD donors). In our cohort, HOPE was associated with lower rates of EAD, primary graft non function (PGNF) and Grade ≥ 3b postoperative complications. The survival analysis in the present study was limited by a relatively short follow‐up; however, more evidence is gathering in literature confirming the beneficial impact of HOPE even in the long‐term [25].

The potential of MP extends beyond graft preservation. It also enables viability assessment, which has facilitated successful transplantation of organs previously deemed unfit; various viability criteria have been proposed although none is yet universally accepted [26]. This has led to an overall reduction in discard rates (up to 30%), although specific results are difficult to generalize due to the different policies applied in each center [5, 27, 28]. Two trials have demonstrated the feasibility of LT of MP treated grafts that would have been otherwise discarded [29, 30]. However, due to practical and ethical issues, proper RCTs and solid evidence are still lacking. In our center, the use of discarded grafts increased over time. The main motives for rejecting a graft were low graft quality (steatosis > 40%) and high medical, infective, or oncological risk donors. HOPE was mainly performed at the surgeon's discretion for discarded grafts. However, with time and more experience, we observed a shift toward a more routine application of HOPE for marginal grafts in our cohort. Initially encouraged by the results of the aforementioned randomized trial, our approach is also driven by the positive outcomes after LT of ever more marginal grafts treated with HOPE. In fact, despite the lower quality (or higher DRI), marginal, including discarded grafts, treated with HOPE have consistently shown good, acceptable, noninferior outcomes compared to the other subgroups. While our results are limited by the small and heterogeneous sample of discarded grafts, they are in line with the most recent reports on the matter. As has often been the case with HOPE, its perceived advantages and utilization in clinical practice have preceded the academic work sustaining its application. We strongly believe that future multicenter and prospective trials shall confirm our findings and expand the pool of available organs that can be transplanted safely with HOPE.

Lastly, the quick diffusion of MP has also led to questions on its routine application in LT [31]. Few studies have addressed this issue with mixed results. Evidence of HOPE benefit in standard criteria donors remains scarce. An increase of MP for standard criteria was observed also in our Center. While more data and experience are gathered [32], a future with routine MP can be envisaged.

The main limitations of our study include its retrospective nature and bias owed to the nonrandomized application of HOPE, as well as the relatively contained number of DCDs, the lack of cost analysis, and the limited long‐term follow‐up. On the other hand, the strongest point is the large cohort and results from real‐world evidence from a center with extensive experience with HOPE.

In conclusion, our findings highlight how the implementation of HOPE has enabled the safe and effective use of high‐risk liver grafts, resulting in excellent post‐transplant outcomes. With efficient clinical and surgical management, HOPE represents a powerful tool in expanding the donor pool and improving graft viability in contemporary LT.

## Author Contributions

Research design: R.M., C.M., S.A., M.M.C., D.A. Writing of the paper: D.G., F.G., G.G., S.M., R.M. Performance of the research: B.C., P.E., O.F., M.L. Analytic tools: G.G., D.G.M., E.D.P., Z.C., L.A. Data analysis: D.G., F.G., P.E.

## Funding

The work reported in this publication was funded by the Italian Ministry of Health, RC‐2026‐2801326.

## Disclosure

The authors have nothing to report.

## Conflicts of Interest

The authors declare no conflicts of interest.

## Supporting information


**Table S1:** Uni‐ and multivariate risk factor for EAD in ECD donors.
**Table S2:** Logistic regression for graft loss at 12 months after liver transplantation in ECD donors.

## Data Availability

Data supporting the findings of this study are available from the corresponding author on request according to national and international legislation regarding privacy and data protection.
