# Supplementary material for: Pushing the Limit for Marginal Grafts in the Era of Machine Perfusion: A Tale of HOPE From a Leading Italian Institution
Source: Artif Organs. 2026 Apr 28;50(7):1027–36. doi: 10.1111/aor.70137 (PMC13397262; doi:10.1111/aor.70137)
Supplement: Supplementary file 1 — Table S1: Uni‐ and multivariate risk factor for EAD in ECD donors. Table S2: Logistic regression for graft loss at 12 months after liver transplantation in ECD donors. [file AOR-50-1027-s001.docx]

| **Supplementary Table 1 Uni- and multivariate risk factor for EAD in ECD donors** | | | | |
| --- | --- | --- | --- | --- |
|  | **Univariate analysis** | | **Multivariate analysis** | |
|  | **OR (95% CI)** | **p** | **OR (95% CI)** | **p** |
| Recipient age | 1.0 (1.0 – 1.0) | 0.4 | - | - |
| Recipient male sex | 1.1 (0.7 – 1.7) | 0.8 | - | - |
| Recipient BMI | 1.0 (0.9 – 1.0) | 0.7 | - | - |
| MELD at transplant | 1.0 (1.0 – 1.1) | 0.2 | - | - |
| Donor age | 1.0 (1.0 – 1.0) | 0.8 | - | - |
| Donor male sex | 1.3 (0.9 – 2.1) | 0.2 | - | - |
| Donor BMI | 1.0 (0.9 – 1.0) | 0.8 | - | - |
| Macrosteatosis | 1.02 (1.00 – 1.05) | 0.041 | - | - |
| Donor Risk Index | 0.6 (0.3 – 1.1) | 0.082 | - | - |
| Cold Ischemia Time | 1.00 (1.00 – 1.01) | 0.001 | 1.00 (1.00 – 1.01) | 0.000 |
| HOPE | 0.54 (0.35 – 0.84) | 0.007 | 0.50 (0.32 – 0.80) | 0.003 |
| **EAD – Early Allograft Dysfunction, ECD – Extended Criteria Donors, MELD – Model for End-stage Liver Disease, BMI – Body Mass Index, HOPE – Hypothermic Oxygenated Perfusion** | | | | |

| **Supplementary Table 2** **Logistic regression for graft loss at 12 months after liver transplantation in ECD donors** | | | |
| --- | --- | --- | --- |
|  | **OR** | **95% CI** | **P** |
| Other ECDs – SCS | -- | -- | -- |
| Other ECDs – HOPE | 0.812 | 0.376 – 1.753 | 0.6 |
| DCD – HOPE | 1.394 | 0.590 – 3.297 | 0.4 |
| Discarded – SCS | 1.534 | 0.473 – 1.974 | 0.5 |
| Discarded – HOPE | 1.185 | 0.411 – 3.415 | 0.8 |
| **ECD – Extended Criteria Donors; SCS – Static Cold Storage; HOPE – Hypothermic oxygenated perfusion, DCD – Donor from Cardiac Death** | | | |

**STROBE CHECKLIST**

|  | **Item No** | **Recommendation** | **Page No** |
| --- | --- | --- | --- |
| **Title and abstract** | 1 | (*a*) Indicate the study’s design with a commonly used term in the title or the abstract | 3 |
|  |  | (*b*) Provide in the abstract an informative and balanced summary of what was done and what was found | 6 |
| **Introduction** | | | |
| Background/rationale | 2 | Explain the scientific background and rationale for the investigation being reported | 7 |
| Objectives | 3 | State specific objectives, including any prespecified hypotheses | 7 |
| **Methods** | | | |
| Study design | 4 | Present key elements of study design early in the paper | 7 |
| Setting | 5 | Describe the setting, locations, and relevant dates, including periods of recruitment, exposure, follow-up, and data collection | 7 |
| Participants | 6 | (*a*) Give the eligibility criteria, and the sources and methods of selection of participants. Describe methods of follow-up | 7-8 |
|  |  | (*b*) For matched studies, give matching criteria and number of exposed and unexposed |  |
| Variables | 7 | Clearly define all outcomes, exposures, predictors, potential confounders, and effect modifiers. Give diagnostic criteria, if applicable | 7-8 |
| Data sources/ measurement | 8* | For each variable of interest, give sources of data and details of methods of assessment (measurement). Describe comparability of assessment methods if there is more than one group | 7-8 |
| Bias | 9 | Describe any efforts to address potential sources of bias | 7-8 |
| Study size | 10 | Explain how the study size was arrived at | 7-8 |
| Quantitative variables | 11 | Explain how quantitative variables were handled in the analyses. If applicable, describe which groupings were chosen and why | 7-8 |
| Statistical methods | 12 | (*a*) Describe all statistical methods, including those used to control for confounding | 8 |
|  |  | (*b*) Describe any methods used to examine subgroups and interactions | 8 |
|  |  | (*c*) Explain how missing data were addressed | 8 |
|  |  | (*d*) If applicable, explain how loss to follow-up was addressed | 8 |
|  |  | (*e*) Describe any sensitivity analyses | 8 |
| **Results** | | |  |
| Participants | 13* | (a) Report numbers of individuals at each stage of study—eg numbers potentially eligible, examined for eligibility, confirmed eligible, included in the study, completing follow-up, and analysed | 8-9 |
|  |  | (b) Give reasons for non-participation at each stage | 8-9 |
|  |  | (c) Consider use of a flow diagram | Na |
| Descriptive data | 14* | (a) Give characteristics of study participants (eg demographic, clinical, social) and information on exposures and potential confounders | 9 |
|  |  | (b) Indicate number of participants with missing data for each variable of interest | 9-10 |
|  |  | (c) Summarise follow-up time (eg, average and total amount) | 10 |
| Outcome data | 15* | Report numbers of outcome events or summary measures over time | 9-10 |

| Main results | 16 | (*a*) Give unadjusted estimates and, if applicable, confounder-adjusted estimates and their precision (eg, 95% confidence interval). Make clear which confounders were adjusted for and why they were included | 9-10 |
| --- | --- | --- | --- |
|  |  | (*b*) Report category boundaries when continuous variables were categorized | 9-10 |
|  |  | (*c*) If relevant, consider translating estimates of relative risk into absolute risk for a meaningful time period | 9-10 |
| Other analyses | 17 | Report other analyses done—eg analyses of subgroups and interactions, and sensitivity analyses | 10 |
| **Discussion** | | | |
| Key results | 18 | Summarise key results with reference to study objectives | 10-11 |
| Limitations | 19 | Discuss limitations of the study, taking into account sources of potential bias or imprecision. Discuss both direction and magnitude of any potential bias | 13 |
| Interpretation | 20 | Give a cautious overall interpretation of results considering objectives, limitations, multiplicity of analyses, results from similar studies, and other relevant evidence | 11-13 |
| Generalisability | 21 | Discuss the generalisability (external validity) of the study results | 12-13 |
| **Other information** | | | |
| Funding | 22 | Give the source of funding and the role of the funders for the present study and, if applicable, for the original study on which the present article is based | 4 |
